# Early clinical efficacy of hip hemiarthroplasty via the SuperCap approach vs. the posterolateral approach in elderly patients with femoral neck fractures: a randomized controlled trial

**DOI:** 10.3389/fsurg.2026.1922436

**Published:** 2026-08-05

**Authors:** Jiquan Shen, Changjian Zhou, Xinggao Wang, Yuxuan Chen, Run Liu, Bo Wang

**Affiliations:** 1 Department of Orthopaedics, The First Affiliated Hospital of Lishui University, Lishui Hospital of Wenzhou Medical University, Lishui People’s Hospital, Lishui, Zhejiang, China; 2 Department of Orthopedic Surgery, The Second Affiliated Hospital, Zhejiang University School of Medicine, Hangzhou, China

**Keywords:** early recovery, elderly, femoral neck fractures, hip hemiarthroplasty, minimally invasive surgery, posterolateral approach, SuperCap approach

## Abstract

**Objective:**

To evaluate the clinical efficacy, perioperative trauma, and early functional recovery trajectory of hip hemiarthroplasty (HA) via the SuperCap approach vs. the traditional posterolateral approach (PLA) in patients aged ≥75 years with displaced femoral neck fractures (FNFs).

**Methods:**

A prospective, single-center, randomized controlled trial was conducted between July 2023 and January 2024, initially recruiting 52 geriatric patients with displaced FNFs (Garden types III and IV). Forty-eight patients (24 per group) completed the 6- and 12-month functional assessments. The primary outcome was the Harris Hip Score (HHS) at 6 months postoperatively. Secondary outcomes encompassed perioperative metrics, early mobility indices [Time to Mobilization, Timed Up and Go (TUG)], pain Visual Analog Scale (VAS), laboratory biomarkers of muscle trauma and inflammation [e.g., creatine kinase (CK), hemoglobin (Hb)], radiographic positioning, and complications.

**Results:**

The primary outcome (6-month HHS) showed no statistically significant difference between groups (91.04 ± 1.46 vs. 90.67 ± 1.97; mean difference 0.37; 95% CI −0.64 to 1.38; *P* = 0.466). However, the SuperCap cohort yielded significantly superior early functional recovery, evidenced by higher HHS and lower VAS scores up to 3 months postoperatively (*P* < 0.05). The SuperCap group demonstrated superior perioperative metrics, including shorter operative time (35.79 ± 4.19 vs. 50.71 ± 5.18 min), smaller incisions (6.03 ± 0.54 vs. 9.75 ± 1.07 cm), reduced intraoperative blood loss (87.50 ± 27.70 vs. 139.17 ± 36.23 mL), and shorter hospital stays (all *P* < 0.05). Postoperative day-1 Hb was significantly higher, and CK levels were significantly lower in the SuperCap group (*P* < 0.05), consistent with reduced muscle trauma. SuperCap patients also exhibited faster Time to Mobilization and shorter TUG duration. Radiographic component positioning and complication rates were comparable between groups.

**Conclusion:**

While achieving comparable short-term functional outcomes to the traditional PLA, the SuperCap approach significantly minimizes skeletal muscle trauma, reduces perioperative blood loss, and accelerates early functional rehabilitation. It serves as a highly effective, tissue-sparing option that facilitates rapid postoperative recovery in vulnerable geriatric patients.

**Clinical Trial registration:**

Chinese Clinical Trial Registry, ChiCTR2300073391.

## Introduction

Femoral neck fracture (FNF) is a prevalent hip injury among the elderly population, accounting for approximately 53% of all proximal femoral fractures. Driven by extended life expectancy and global population aging, its incidence continues to escalate and is projected to surpass 6.3 million cases annually by 2050 (1, 2). Elderly FNF patients frequently present with osteoporosis and multiple comorbidities, leading to high postoperative complication rates (3). Consequently, functional recovery is often compromised, with disability rates reaching 30%–50% and 1-year mortality rates peaking at 20%–30%, severely diminishing patients' quality of life (4–6). Artificial hip replacement is currently the gold standard for treating displaced FNFs (Garden types III–IV) in this demographic, offering rapid pain relief and functional restoration (7, 8). Specifically, hip hemiarthroplasty (HA) is widely recommended as the primary intervention for elderly patients with limited pre-injury mobility and shorter life expectancies (9, 10).

Traditionally, HA utilizes the posterolateral approach (PLA) due to its excellent anatomical exposure and technical familiarity. However, the requisite detachment of the short external rotators and posterior joint capsule may increase the risks of intraoperative blood loss, postoperative joint instability, and prosthetic dislocation (11). To mitigate these complications, minimally invasive techniques have gained prominence, offering reduced bleeding, lower infection rates, and preservation of the abductor and external rotator musculature (12). The SuperCap (SuperCapsular) approach, initially introduced by Murphy in 2003, exemplifies a true tissue-sparing, intermuscular technique. Utilizing a 6–8 cm incision (which may be shortened to 5–7 cm with increasing surgeon experience) at the apex of the greater trochanter, it accesses the hip joint through the natural interval between the piriformis and the gluteus minimus. This approach preserves the external rotators and posterior capsule intact, minimizing soft-tissue trauma (13). Theoretically, this approach may reduce skeletal muscle damage, limit perioperative blood loss, improve early postoperative pain control, and accelerate early rehabilitation.

Despite growing interest in this technique, the available evidence remains limited in one clinically important subgroup: patients aged ≥75 years undergoing HA specifically for displaced FNFs. Most previous comparative studies have enrolled younger elderly populations (e.g., ≥60 or ≥65 years), included mixed arthroplasty procedures such as total hip arthroplasty and HA, or provided limited prospective randomized data focused exclusively on very old patients. Yet this population is particularly vulnerable to perioperative stress, delayed mobilization, and bedrest-related complications such as pneumonia, pressure ulcers, and venous thromboembolism (14, 15).

Accordingly, the present prospective randomized controlled trial aimed to compare the SuperCap approach with the traditional PLA in patients aged ≥75 years undergoing HA for displaced FNFs. By integrating perioperative, functional, biochemical, and radiographic assessments, we sought to determine whether the tissue-sparing characteristics of the SuperCap approach translate into measurable early clinical benefits in this high-risk geriatric population.

## Methods

### Study design and ethical approval

This was a prospective, single-center, randomized controlled trial. The study protocol was approved by the Medical Ethics Committee of Lishui People's Hospital (approval No. 022-01) on July 3, 2023 and was prospectively registered in the Chinese Clinical Trial Registry (ChiCTR2300073391) on July 10, 2023. The first patient was enrolled on July 15, 2023, after trial registration. The study design and reporting adhered to the Consolidated Standards of Reporting Trials (CONSORT) guidelines and the Declaration of Helsinki (as revised in 2013). Written informed consent was obtained from all patients or their legal representatives prior to enrollment.

### Inclusion and exclusion criteria

Inclusion criteria: (1) fresh, completely displaced FNFs (Garden types III and IV); (2) age ≥75 years; (3) scheduled to undergo HA via either the SuperCap or PLA; (4) sufficient cognitive and physical capability to complete the 12-month postoperative follow-up.

Exclusion criteria: (1) pathological fractures, pre-operative osteonecrosis of the femoral head, or advanced osteoarthritis of the hip; (2) severe preexisting anatomical deformities or hip dysfunction prior to the fracture; (3) severe obesity (BMI > 35 kg/m^2^); (4) inability to complete the 12-month follow-up due to limited life expectancy or severe comorbidities. For clarity, severe comorbidity in this study referred to serious medical conditions likely to preclude surgery, postoperative rehabilitation, or protocolized follow-up, including active malignancy under treatment, advanced dementia, end-stage renal disease, severe heart failure, severe chronic respiratory insufficiency, severe uncontrolled diabetes, or comparable conditions.

### Sample size calculation and randomization

The sample size was powered to detect a minimal clinically important difference (MCID) in the HHS at 6 months postoperatively, which served as the primary endpoint. Based on prior literature (15–17), which suggested that functional differences between approaches converge by 6 months, we sought to detect a conservative mean difference of 3 points with a Standard Deviation (SD) of 3. This required a minimum of 23 patients per group to achieve 90% power (1−*β* = 0.90) at a two-sided *α* = 0.05. This small expected difference reflects our hypothesis of functional equivalence at the primary endpoint rather than superiority. Accounting for an anticipated 10% dropout rate, the target was set at 26 patients per group (total *n* = 52).

**Figure 1:**
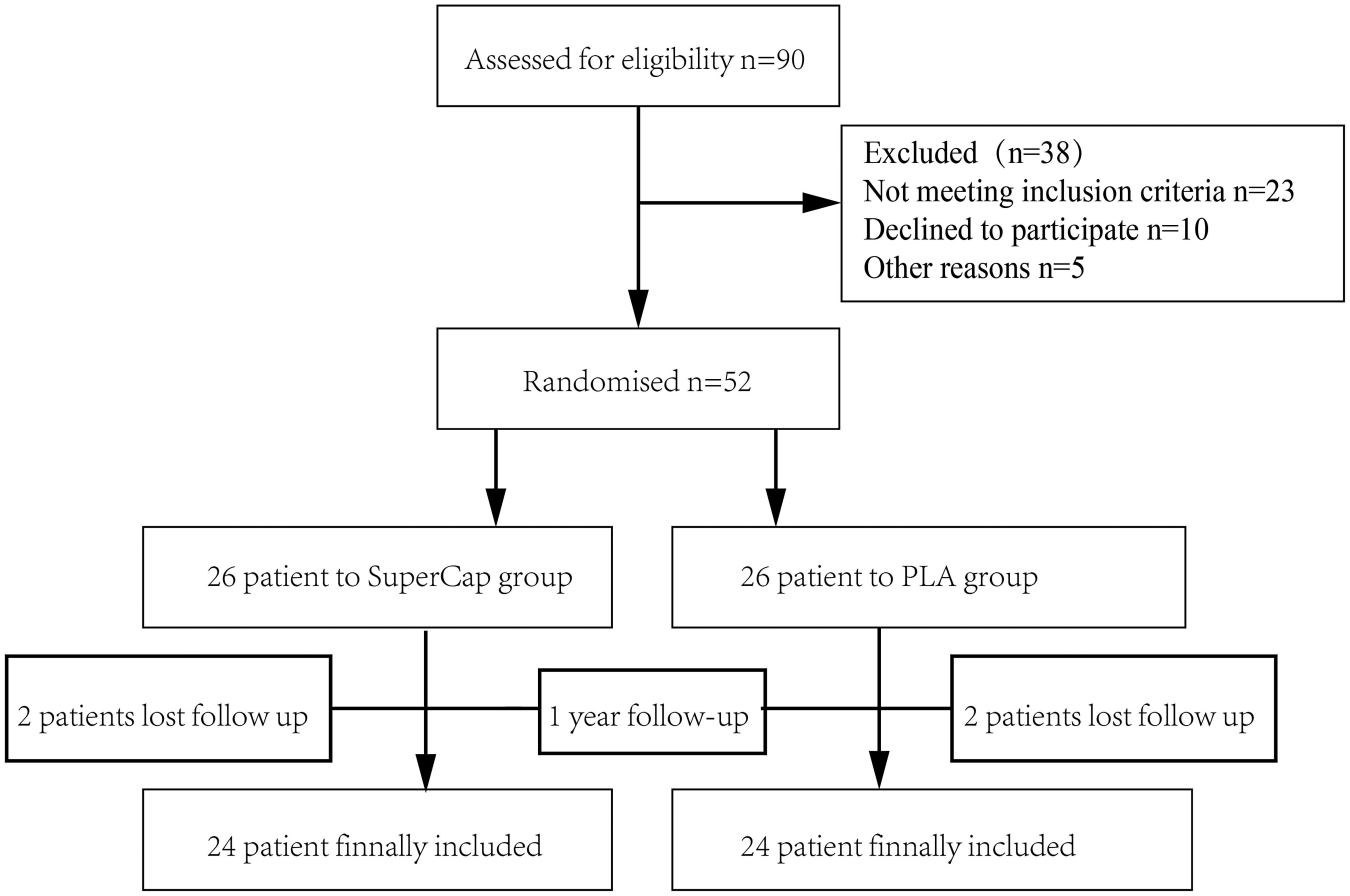
CONSORT flow diagram illustrating the progress of participants through the phases of the randomized controlled trial.

Between July 2023 and January 2024, 90 consecutive patients presenting with FNFs were screened. Ultimately, 52 eligible patients were enrolled and randomized in a 1:1 ratio into the SuperCap group (*n* = 26) or the PLA group (*n* = 26) using a computer-generated, simple randomization sequence. Allocation concealment was maintained via sequentially numbered, opaque, sealed envelopes opened by the surgical coordinator immediately prior to surgery. The CONSORT flow diagram is shown in Figure 1.

### Blinding

Given the visible difference in incision length, full blinding was not feasible. The operating surgeon was necessarily aware of the allocated approach. Patients were not blinded to allocation, which is acknowledged as a potential source of expectation bias affecting patient-reported outcomes (VAS, HHS subcomponents). Outcome assessors (one research fellow and one postgraduate student) responsible for functional scoring, laboratory data collation, and radiographic measurement were blinded to allocation. Statistical analyses were performed by an independent statistician blinded to group allocation.

### Surgical techniques

All surgeries were performed by the same senior orthopedic surgeon, who was proficient in hip arthroplasty. All patients received combined spinal-epidural anesthesia to standardize the anesthetic technique. The hemiarthroplasty implants utilized were the SuperCap System (MicroPort Orthopedics, Suzhou, China), standard bipolar/unipolar stems (Biomet Orthopedics, USA), and the Chunli Medical bipolar/unipolar hip prosthesis (Beijing Chunlizhengda Medical Instruments Co., Ltd., Beijing, China).

#### SuperCap approach

**Figure 2:**
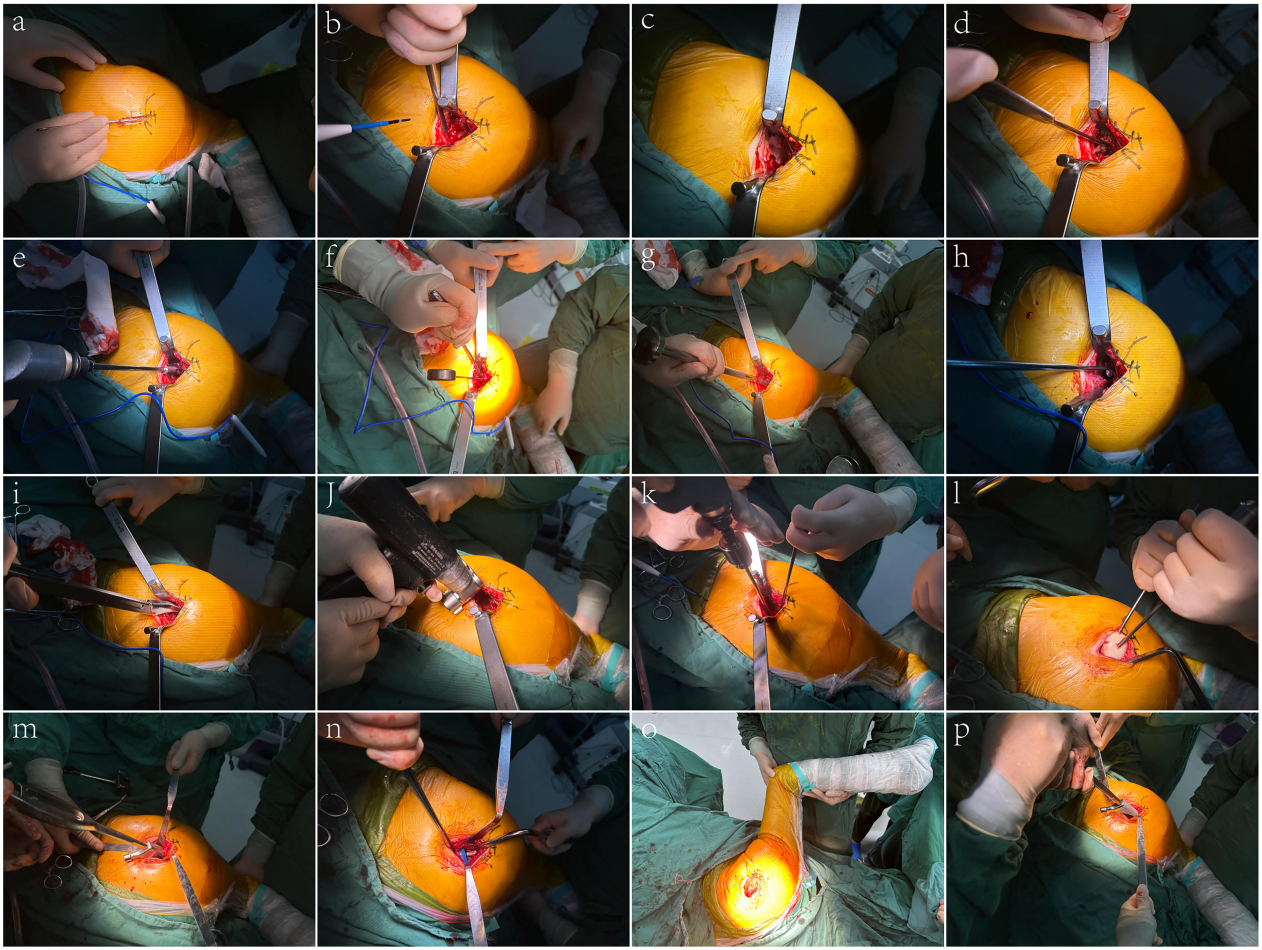
The SuperCap approach for Hip hemiarthroplasty (HA) for femoral neck fractures. **(a)** Patient positioning and skin incision; **(b)** Splitting of the gluteus maximus fascia; **(c)** Inserting two blunt Hohmann retractors anterior and posterior to the joint capsule; **(d)** Incising the joint capsule to expose the femoral neck; **(e)** Opening the proximal femoral medullary canal with a starter awl; **(f)** Insertion of the femoral alignment guide pin; **(g)** Creation of the entry slot with a round calcar punch to define stem anteversion; **(h)** Clearance of proximal cancellous bone with a calcar curette; **(i)** Sequential *in situ* broaching; **(j)** *in situ* femoral neck osteotomy using the trial broach as a reference; **(k)** Insertion of two Schanz pins into the femoral head for controlled rotation and extraction; **(l)** Removal of the femoral head; **(m)** Inserting the femoral prosthesis; **(n)** Completing the prosthesis assembly; **(o)** Assessment of prosthesis position and stability, including fluoroscopic confirmation when required; **(p)** Final implantation.

The patient was placed in the standard lateral decubitus position with the operative hip slightly adducted, flexed 45°–60°, the knee flexed to 90°, and the limb internally rotated 20°–30° (Figure 2a). A 5–7 cm skin incision was made proximally from the tip of the greater trochanter along the longitudinal axis of the femur (Figure 2a). The gluteus maximus fascia was incised in line with its fibers (Figure 2b). Using blunt finger dissection, the interval between the piriformis and gluteus minimus was identified, and the posterior border of the gluteus medius was retracted anteriorly. Blunt Hohmann retractors were placed anterior and posterior to the capsule (Figure 2c), followed by a superior capsulotomy extending from the saddle of the femoral neck to approximately 1 cm proximal to the acetabular rim, thereby exposing the intracapsular structures (Figure 2d). Importantly, the short external rotators remained intact. After opening the femoral canal at its zenith, a starter awl was first used to establish the intended direction of the canal (Figure 2e), followed by insertion of a femoral alignment guide pin toward the femoral condyles for orientation (Figure 2f). According to the direction of the guide pin, a metaphyseal reamer was used to enlarge the proximal femoral canal. A round calcar punch was then used to create the entry slot (Figure 2g) in order to determine stem anteversion, and the preparation was extended from the canal entry point along the saddle of the femoral neck toward the acetabular rim. Subsequently, a calcar curette was used to carefully remove cancellous bone from the medial and lateral aspects of the proximal canal (Figure 2h), and sequential broaching was performed *in situ* to the appropriate size (Figure 2i). Because all femoral-side preparation was completed *in situ*, without excessive adduction, external rotation, or forceful levering of the proximal femur, stress concentration on the greater trochanter and proximal femur could be reduced, thereby decreasing the risk of iatrogenic fracture.

Once the selected trial broach had been seated, an *in situ* femoral neck osteotomy was performed with an oscillating saw using the top of the broach as the reference (Figure 2j). For removal of the fractured femoral head, the residual femoral neck was first trimmed with the oscillating saw, after which a first Schanz pin was inserted into the femoral head to initiate controlled rotation, followed by insertion of a second Schanz pin to enhance purchase and rotational control (Figure 2k). By holding both pins simultaneously, the surgeon gently rotated the femoral head to detach it from the ligamentum teres and extracted it through the superior window (Figure 2l). After removal of the trial broach, femoral stem anteversion and prosthetic alignment were assessed using the orientation of the intramedullary alignment rod together with the direction of the patella indicated by the assistant (Figure 2m), after which the appropriately sized bipolar head was assembled (Figure 2n). Full range-of-motion testing was performed to assess implant and compatibility (Figure 2o). During the initial adoption phase of the technique, fluoroscopy was used as needed to confirm canal preparation and trial component orientation; after the learning curve had been overcome, fluoroscopy was generally performed only once before definitive implantation to verify stem alignment and prosthesis position. The final prosthesis was then implanted (Figure 2p). After reconfirmation of stability, the capsule was meticulously repaired, followed by routine layered wound closure.

#### Posterolateral approach (PLA)

With the patient in the lateral decubitus position, a standard 9–11 cm longitudinal incision was made, centered over the posterior aspect of the greater trochanter. The gluteus maximus was split bluntly, and the short external rotators (including the piriformis) were identified, tagged, and released at their insertions, along with the posterior joint capsule, to expose the hip joint. After performing a standard femoral neck osteotomy, the hip was dislocated posteriorly, the fractured head was removed, and standard canal preparation and prosthesis implantation were performed. Upon achieving stable reduction, the posterior capsule and external rotators were meticulously sutured back to the greater trochanter via transosseous tunnels. The wound was then closed in layers.

### Perioperative management and rehabilitation protocol

A standardized enhanced recovery after surgery (ERAS) protocol was applied to both groups, including identical multimodal analgesia (intravenous parecoxib, oral celecoxib, and patient-controlled analgesia within 48 h), thromboprophylaxis (oral rivaroxaban 10 mg once daily; Bayer AG, Leverkusen, Germany), and transfusion criteria (hemoglobin < 80 g/L). The postoperative rehabilitation protocol was identical in both groups and was supervised by the same physiotherapy team: ankle-pumping exercises and bed-edge sitting on postoperative day (POD) 0; assisted standing with a walker on POD 1–3; then partial weight-bearing ambulation as tolerated; and progressive resistance and balance training thereafter. The decision on first ambulation was based solely on patient tolerance and pain control, independent of the surgical group.

### Outcome measures and data collection

#### Baseline patient characteristics

Upon admission, comprehensive demographic and baseline clinical data were recorded, including age, sex, affected side, body mass index (BMI), and the American Society of Anesthesiologists (ASA) classification. These variables are presented descriptively to characterize the randomized cohorts at baseline.

#### Functional assessment

Hip function was assessed using the HHS, evaluated prospectively at 1 week, and 1, 3, 6, and 12-months postoperatively.

#### Secondary outcome measures

Secondary endpoints encompassed perioperative parameters, laboratory biomarkers, early functional mobility, pain relief, and radiographic component positioning.

Perioperative Parameters: General surgical metrics included operative time, intraoperative blood loss, incision length, length of hospital stay (LOS), allogeneic blood transfusion rates, and the incidence of any surgery-related complications.

Clinical and Early Functional Outcomes: Postoperative pain was dynamically assessed using the VAS at admission; on PODs 1, 3, and 7; and at 1, 3, 6, and 12-months postoperatively. Early functional mobility was quantified using two distinct metrics: “Time to Mobilization” (defined as the number of days elapsed from the completion of surgery to the patient's first assisted or unassisted step) and the TUG test (the time required to rise from an armchair, walk 3 m, turn around, and sit back down).

Laboratory Biomarkers: To quantitatively evaluate surgical trauma and physiological responses, serial venous blood samples were analyzed with strict adherence to specific timeframes: Hb levels, indicative of both total and occult (hidden) blood loss, were measured on the day of admission (preoperatively), and on PODs 1 and 3. Acute systemic inflammatory markers (CRP and ESR) and serum CK levels (serving as a biochemical indicator for acute skeletal muscle injury) were monitored on the day of admission, and systematically on PODs 1, 3, and 7.

**Figure 3:**
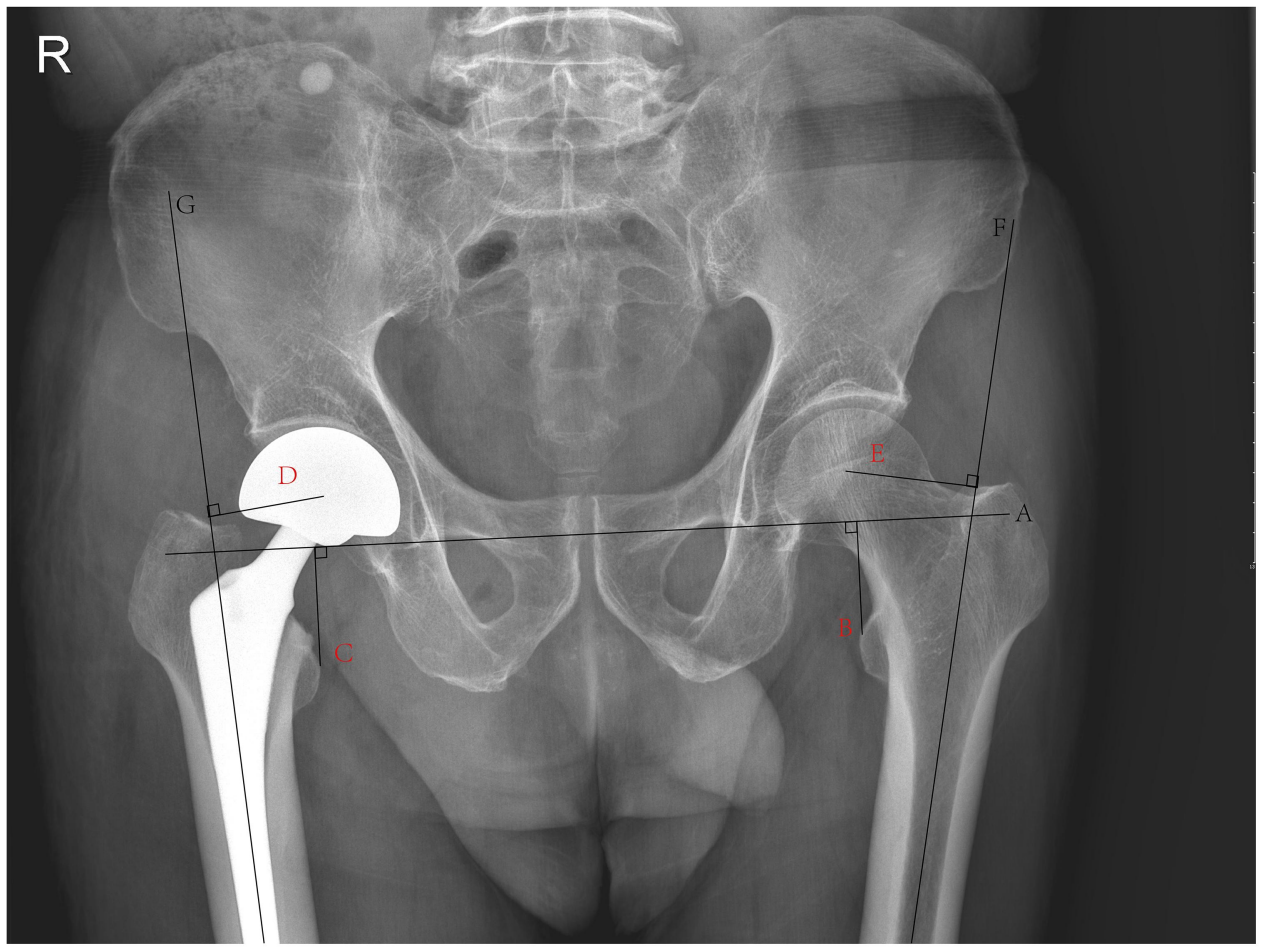
Postoperative anteroposterior pelvic radiograph demonstrating radiographic precision of component positioning [horizontal femoral offset (HFO) and leg length discrepancy (LLD)]. A, inter-teardrop line; B, distance from the most prominent point of the lesser trochanter on the healthy side to line A; C, distance from the most prominent point of the lesser trochanter on the operated side to line A; D, offset on the operated side; E, offset on the healthy side; G, femoral shaft bisector line on the operated side; F, femoral shaft bisector line on the healthy side.

Radiographic Evaluation: Standardized anteroposterior pelvic radiographs (obtained with the patient supine and the x-ray beam centered on the pubic symphysis with a 100-cm focal distance) were taken within one week postoperatively. Using the hospital's Picture Archiving and Communication System (PACS), two critical parameters were measured: HFO, defined as the perpendicular distance from the center of the prosthetic head to the anatomical axis of the femur; and LLD, assessed by the difference in the perpendicular distances from the most prominent aspect of the bilateral lesser trochanters to the standard bi-teardrop line (Figure 3).

### Statistical analysis

Analyses were performed using SPSS version 22.0 (SPSS Inc., Chicago, IL, USA). Normality was assessed by the Shapiro–Wilk test. Normally distributed continuous variables were expressed as mean ± SD and compared using independent-samples *t*-tests; non-normally distributed variables were expressed as median (IQR) and compared using the Mann–Whitney *U* test. Categorical variables were compared using Chi-square or Fisher's exact tests. For repeated measures (VAS, HHS, Hb, CRP, ESR, CK), a mixed-design repeated-measures ANOVA was used, with group as the between-subjects factor and time as the within-subjects factor; group × time interaction and main effects were reported. Sphericity was assessed by Mauchly's test, with Greenhouse-Geisser correction applied where appropriate. Pairwise comparisons at individual time points were Bonferroni-corrected. For each between-group comparison, mean differences with 95% confidence intervals (CIs) and effect sizes (Cohen's *d* for continuous variables) are reported in addition to *P* values. A two-sided *P* < 0.05 was considered statistically significant.

## Results

### Study population and follow-up

A total of 52 patients were randomized (26 per group). All 52 patients underwent their allocated procedure and were included in perioperative, in-hospital laboratory, and safety analyses. During the 12-month follow-up, 4 patients were lost to functional outcome assessment: 2 patients in the SuperCap group and 2 patients in the PLA group were lost to follow-up due to relocation or withdrawal of consent. Accordingly, 48 patients (24 per group) completed the 6- and 12-month functional assessments and constituted the per-protocol population for functional outcome analyses.

### Baseline characteristics

The SuperCap group comprised 24 patients who completed follow-up (10 male, 14 female; 10 left, 14 right; Garden III: 8, Garden IV: 16), and the PLA group comprised 24 patients (11 male, 13 female; 12 left, 12 right; Garden III: 10, Garden IV: 14). Baseline characteristics were descriptively similar between the two groups. Mean age was 84.83 ± 4.84 years in the SuperCap group and 82.71 ± 6.66 years in the PLA group; mean BMI was 21.02 ± 3.00 and 22.83 ± 2.37 kg/m^2^, respectively; and ASA classification was predominantly grade II–III in both cohorts (Table 1).

**Table 1:** The characteristics of patients in two groups (mean ± SD).

Parameters	SuperCap group	PLA group
No. of patients	24	24
Age (years)	83.83 ± 4.84	82.71 ± 6.66
Gender(M/F)	10/14	11/13
Side (L/R)	10/14	12/12
Garden (III/IV)	8/16	10/14
BMI (kg/m^2^)	21.02 ± 3.00	22.83 ± 2.37
ASA (grade)	2.46 ± 0.51	2.33 ± 0.48

PLA, posterolateral approach; M, male; F female; L, left; R, right; BMI, body mass index; ASA, American Society of Anesthesiologists.

### Perioperative parameters and length of stay

The SuperCap group demonstrated markedly superior perioperative metrics, confirming its minimally invasive nature. The incision length was reduced by nearly 40% in the SuperCap group compared to the PLA group (6.03 ± 0.54 vs. 9.75 ± 1.07 cm, mean difference −3.72 cm; 95% CI −4.21 to −3.23; Cohen's *d* = 4.38; *P* < 0.001). Consequently, intraoperative blood loss was significantly lower in the SuperCap cohort (87.50 ± 27.97 vs. 139.17 ± 36.23 mL, *P* < 0.001). Additionally, the operative time was significantly shorter for the SuperCap procedure (35.79 ± 4.19 vs. 50.71 ± 5.18 min, *P* < 0.001). Despite the disparity in blood loss, the allogeneic blood transfusion rates showed no significant intergroup difference (16.67% vs. 25.00%, *P* = 0.480). Driven by the mitigated surgical trauma, the LOS was significantly shorter in the SuperCap group (8.13 ± 1.68 vs. 9.46 ± 1.91 days, *P* = 0.014) (Table 2).

**Table 2:** The perioperative parameters and length of stay in two groups.

Parameters	SuperCap group	PLA group	*P* value
Operation time (min)	35.79 ± 4.19	50.71 ± 5.18*	0.000
Intraoperative blood loss (mL)	87.50 ± 27.70	139.17 ± 36.23*	0.000
Incision length (cm)	6.03 ± 0.54	9.75 ± 1.07*	0.000
Hospitalization time (days)	8.13 ± 1.68	9.46 ± 1.91*	0.013
Blood transfusion rate (%)	16.67%	25%	0.724

PLA, posterolateral approach.

* *P* < 0.05 indicates significant differences from the PLA group.

### Laboratory biomarker analysis

**Figure 4:**
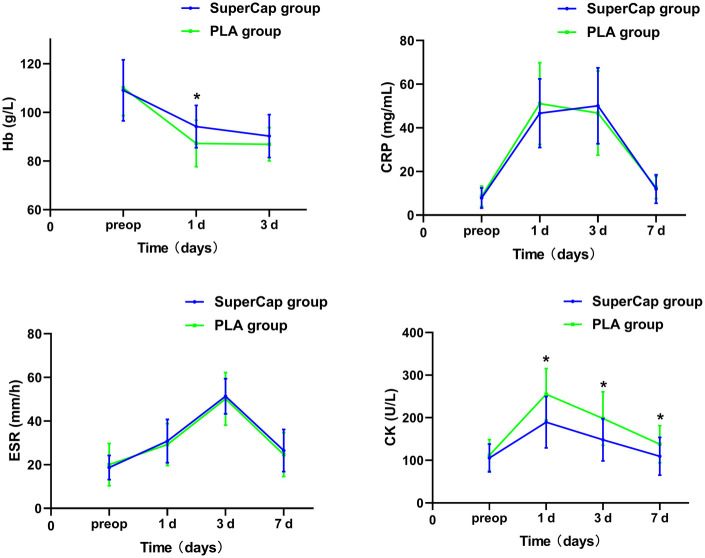
Pre- and postoperative levels of serum markers (Hb, CRP, ESR and CK) in the SuperCap and PLA groups. *Y*-axis represents levels of serum markers and *x*-axis denotes the time points. *P < 0.05 indicates significant differences from the PLA group.

Perioperative hematological analyses corroborated the clinical observations. While preoperative Hb levels were comparable, the Day-1 postoperative Hb level was significantly higher in the SuperCap group (94.13 ± 8.71 vs. 87.20 ± 9.56 g/L, *P* = 0.012), further substantiating the reduction in intraoperative and occult blood loss. Systemic inflammatory responses, quantified by pre- and postoperative CRP and ESR levels, fluctuated similarly between the two cohorts with no statistical disparities (*P* > 0.05). Conversely, serum CK levels were notably attenuated in the SuperCap group. The CK concentrations were significantly lower in the SuperCap group on postoperative Day 1 (189.65 ± 60.50 vs. 255.06 ± 60.32 U/L, *P* < 0.001), Day 3 (148.06 ± 49.76 vs. 198.23 ± 62.78 U/L, *P* = 0.003), and Day 7 (109.55 ± 44.40 vs. 137.80 ± 43.65 U/L, *P* = 0.028), consistent with reduced muscle injury associated with the tissue-sparing technique (Table 3, Figure 4).

**Table 3:** The laboratory biomarker analysis in two groups (mean ± SD).

Parameters	Timings	SuperCap group	PLA group	*P* value
Hb (g/L)	Preop	109.05 ± 12.57	110.09 ± 11.43	0.765
	Day 1	94.13 ± 8.71	87.20 ± 9.56*	0.012
	Day 3	90.23 ± 8.82	86.85 ± 6.81	0.144
CRP (mg/mL)	Preop	7.84 ± 4.64	8.60 ± 4.67	0.573
	Day 1	46.71 ± 15.75	51.07 ± 18.77	0.389
	Day 3	50.10 ± 17.38	46.80 ± 19.30	0.536
	Day 7	11.94 ± 6.53	12.66 ± 5.07	0.671
ESR (mm/h)	Preop	18.69 ± 5.57	20.03 ± 9.67	0.561
	Day 1	30.81 ± 9.96	29.23 ± 9.58	0.579
	Day 3	51.34 ± 8.08	50.19 ± 12.06	0.695
	Day 7	26.52 ± 9.68	24.61 ± 10.06	0.507
CK (U/L)	Preop	105.47 ± 32.83	111.70 ± 36.73	0.538
	Day 1	189.65 ± 60.50	255.06 ± 60.32*	0.000
	Day 3	148.06 ± 49.76	198.23 ± 62.78*	0.004
	Day 7	109.55 ± 44.40	137.80 ± 43.65*	0.031

PLA, posterolateral approach; Hb, hemoglobin; CRP, C-reactive protein; ESR, erythrocyte sedimentation rate; CK, creatine kinase.

* *P* < 0.05 indicates significant differences from the PLA group.

### Clinical function and pain assessment

Early functional mobilization metrics favored the minimally invasive approach. The SuperCap group achieved a significantly faster Time to Mobilization (2.21 ± 0.83 vs. 3.00 ± 0.83 days, *P* = 0.002) and completed the TUG test more quickly (80.79 ± 26.58 vs. 97.37 ± 29.36 s, *P* = 0.045) than the PLA group (Table 4).

**Table 4:** The clinical function and pain assessment in two groups (mean ± SD).

Parameters	Timings	SuperCap group	PLA group	*P* value
TUG (s)	Postop	80.79 ± 26.58	97.37 ± 29.36*	0.046
Time to mobilization (days)	Postop	2.21 ± 0.83	3.00 ± 0.83*	0.002
VAS (score)	Preop	6.21 ± 0.78	6.13 ± 1.36	0.796
	Day 7	3.79 ± 0.88	4.58 ± 1.24*	0.015
	1-month	1.46 ± 0.98	2.38 ± 1.20*	0.007
	3-month	1.04 ± 0.66	1.37 ± 0.77	0.121
	6-month	0.88 ± 0.80	0.96 ± 0.91	0.737
	12-month	0.75 ± 0.79	0.83 ± 0.64	0.690
HHS (score)	Day 7	75.50 ± 4.07	66.12 ± 4.57*	0.000
	1-month	85.17 ± 4.49	79.75 ± 4.83*	0.000
	3-month	90.21 ± 3.26	83.42 ± 4.42*	0.000
	6-month	91.04 ± 1.46	90.67 ± 1.97	0.375
	12-month	91.79 ± 1.14	91.46 ± 1.38	0.333

PLA, posterolateral approach; TUG, timed up and go; VAS, Visual Analog Scale; HHS, Harris Hip Score.

* *P* < 0.05 indicates significant differences from the PLA group.

**Figure 5:**
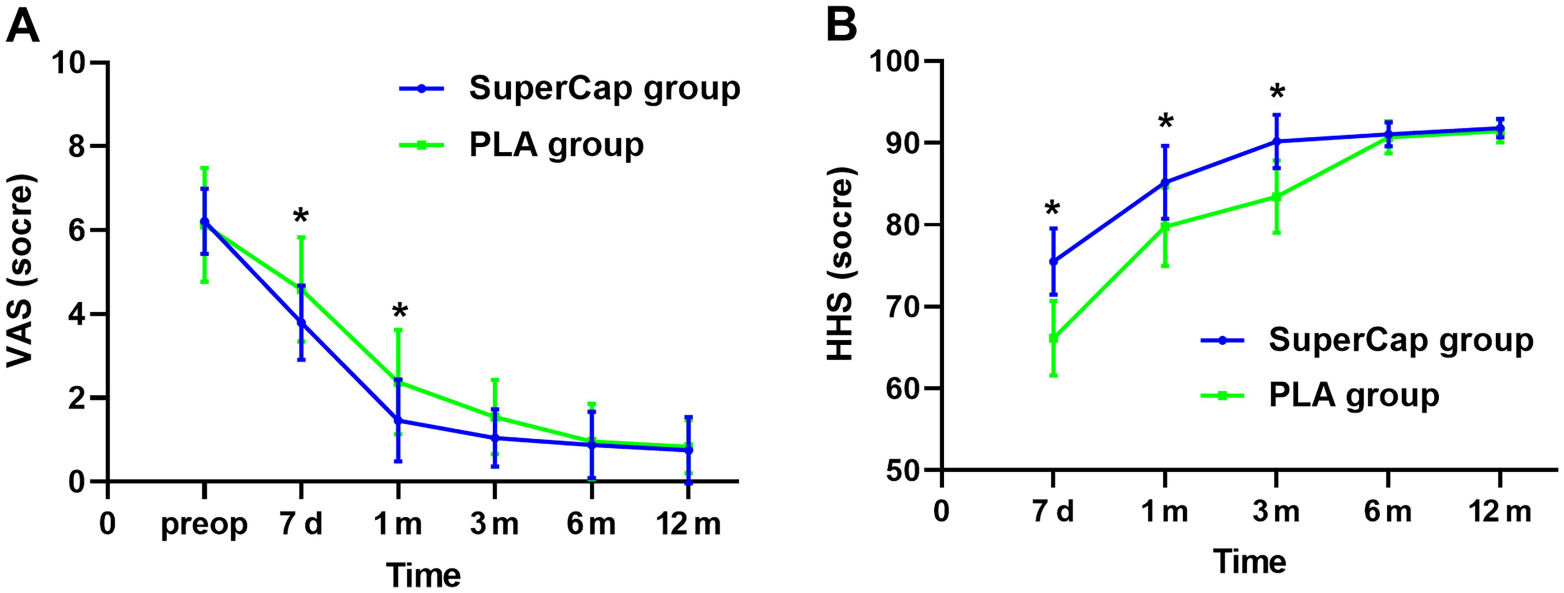
Pre- and postoperative visual analog scale (VAS) **(A)** and Harris hip scores (HHSs) **(B)** in the SuperCap and PLA groups. *Y*-axis represents score and *x*-axis represents denotes the time points. *P < 0.05 indicates significant differences from the PLA group.

Regarding pain management, preoperative VAS scores were equivalent (6.21 ± 0.78 vs. 6.13 ± 1.36, *P* = 0.801). However, the SuperCap group experienced significantly superior pain relief during the early recovery phase: at postoperative Day 7 (3.79 ± 0.88 vs. 4.58 ± 1.24, *P* = 0.015) and 1 month (1.46 ± 0.98 vs. 2.38 ± 1.20, *P* = 0.005). By 3 months postoperatively, VAS scores in both cohorts had converged with no statistical difference (*P* > 0.05) (Table 4, Figure 5A).

Similarly, the Harris Hip Score (HHS) revealed a distinct early advantage for the SuperCap group. Significant intergroup superiority was established as early as postoperative Day 7 (75.50 ± 4.07 vs. 66.12 ± 4.57, *P* < 0.001) and was maintained at 1 month (85.17 ± 4.49 vs. 79.75 ± 4.83, *P* < 0.001) and 3 months (90.21 ± 3.26 vs. 83.42 ± 4.42, *P* < 0.001). As anticipated based on prior literature, the 6-month HHS (91.04 ± 1.46 vs. 90.67 ± 1.97; mean difference 0.37; 95% CI −0.64 to 1.38; Cohen's *d* = 0.21; *P* = 0.466) and 12-month HHS (91.38 ± 1.79 vs. 91.00 ± 2.06, *P* = 0.502) demonstrated convergence, indicating comparable short-term functional restoration (Table 4, Figure 5B).

### Radiographic outcomes

Radiographic evaluations indicated that both surgical approaches fulfilled the biomechanical criteria for accurate component positioning. There were no statistically significant differences between the SuperCap and PLA groups concerning HFO reconstruction (41.61 ± 3.01 vs. 41.95 ± 3.30 mm, *P* = 0.712). Furthermore, LLD was effectively managed within a 1-cm physiological tolerance in both groups (3.12 ± 1.78 vs. 3.62 ± 2.07 mm, *P* = 0.378) (Table 5, Figure 3).

**Table 5:** The radiographic outcomes and complications in two groups.

Parameters	SuperCap group	PLA group	*P* value
Horizontal femoral offset (mm)	41.61 ± 3.01	41.95 ± 3.30	0.710
Leg length discrepancy (mm)	3.12 ± 1.78	3.62 ± 2.07	0.378
DVT rate (%)	4.17%	8.33%	1.000

PLA, posterolateral approach; DVT, Deep vein thrombosis.

### Complications

Safety outcomes were analyzed in all 48 randomized patients. Within the 12-month observation period, no procedure-related deaths occurred. Symptomatic deep vein thrombosis confirmed by Doppler ultrasonography occurred in 1 SuperCap patient (4.17%) and 2 PLA patients (8.33%) (*P* = 1.000). All cases were managed successfully with extended anticoagulation therapy without progression to pulmonary embolism. No periprosthetic fractures, dislocation, deep infections, or pulmonary embolisms were observed in either group. Overall complication rates were comparable between the two groups; however, the study was not powered to detect differences in rare adverse events, and these findings should be interpreted accordingly (Table 5).

## Discussion

The escalating global incidence of FNFs, particularly in patients aged ≥75 years, underscores the imperative for optimized surgical strategies that minimize perioperative morbidity (18). Elderly FNF patients frequently present with complex medical comorbidities (e.g., cardiovascular and metabolic diseases), making prolonged conservative treatment or delayed surgical intervention high-risk due to life-threatening complications (19). Consequently, early surgical restoration of hip function is imperative. Currently, HA is recognized as the optimal surgical modality for displaced FNF in frail elderly patients, as it facilitates immediate postoperative mobilization while minimizing operative time and blood loss (20, 21).

In the pursuit of optimizing hip arthroplasty, minimally invasive techniques aligned with ERAS protocols have gained significant traction (22, 23). While the SuperCap technique was initially conceptualized by Murphy in 2003, its early clinical widespread adoption was largely hindered because it was primarily restricted to hemiarthroplasty. However, the paradigm has shifted dramatically in recent years with the advent of the SuperPATH approach-a synergistic evolution combining the SuperCap technique for femoral preparation with the PATH (Percutaneously Assisted Total Hip) technique for the acetabulum, thereby enabling THA. This technological evolution has catalyzed a robust resurgence of clinical interest in the underlying SuperCap philosophy. By navigating entirely through natural intermuscular planes without the detachment of any tendons, and permitting primary capsular preservation and repair, this technique confers profound dynamic stability, rendering the risk of postoperative dislocation markedly reduced compared to conventional approaches (24). Furthermore, as an ingenious modification derived from the traditional PLA, it retains the familiar anatomical orientation with a shorter learning curve (25), while circumventing the inherent complications frequently associated with the direct anterior approach (DAA), such as lateral femoral cutaneous nerve (LFCN) injury and intraoperative periprosthetic femur fractures (26, 27). Crucially, the SuperCap approach affords surgeons a built-in safety net: should complex intraoperative scenarios arise, the incision can be seamlessly extended to convert into a standard posterior approach, providing unrestricted visualization without compromising patient safety.

It is important to contextualize our outcome findings within the existing evidence base. Multiple prior studies, including the meta-analysis by Ramadanov et al., have demonstrated that functional outcomes following minimally invasive vs. conventional hip arthroplasty approaches typically converge by 6–12 months postoperatively (15, 17). Our finding of equivalent 6- and 12-month HHS scores is therefore consistent with, rather than contradictory to, the established literature. The clinically meaningful question is not whether one approach produces superior long-term function-which is predominantly determined by component positioning, fixation technique, and rehabilitation-but rather whether the minimally invasive approach can deliver the same endpoint via a less traumatic pathway. Our data provide affirmative evidence: the SuperCap approach achieved comparable short-term functional outcomes while significantly reducing perioperative trauma, accelerating early mobilization, and shortening hospitalization-outcomes of particular importance in the frail elderly population vulnerable to bedrest-related complications.

Several previous studies have reported similar short-term benefits of superior capsular tissue-sparing approaches, and these data help contextualize the present findings. Scaglione et al. observed improved functional recovery in elderly patients with femoral neck fractures treated through the SuperCap approach, including better Harris Hip Scores during follow-up, reduced transfusion requirements, smaller incision length, and earlier postoperative weight-bearing (28). In a randomized controlled trial, Jia et al. likewise found that the SuperCap approach significantly reduced early postoperative pain and soft-tissue injury compared with conventional approaches (29). In addition, several Chinese-language studies have described faster early rehabilitation and lower postoperative pain within the first 3 months after surgery (30, 31). In line with these reports, our trial extends the available evidence by focusing specifically on a very elderly population (≥75 years) and by isolating HA rather than combining different arthroplasty procedures.

The minimally invasive superiority of the SuperCap approach was unequivocally demonstrated in our perioperative metrics. Consistent with the intermuscular advantages first described by Murphy, the SuperCap cohort exhibited a nearly 50% reduction in both incision length and intraoperative blood loss compared to the conventional PLA group (13). Furthermore, surgical time and hospital stay were significantly shortened. This enhanced surgical efficiency suggests that bypassing the need for extensive muscular dissection and subsequent tendon repair not only curtails procedural time but fundamentally accelerates the patient's overall biological recovery.

The trajectory of early functional restoration further underscores these benefits. The SuperCap group demonstrated clear superiority in TUG test duration and time to first mobilization, establishing an immediate rehabilitative advantage. At the 1- and 3-month follow-ups, VAS and HHS scores indicated significantly superior pain relief and functional mobility, corroborating the findings of Jia et al. (29). We attribute this early disparity to two primary factors: first, the preservation of the external rotator musculature and posterior capsule mitigates the risk of postoperative capsular contracture; second, the strictly intermuscular navigation minimizes neurovascular stretching and iatrogenic muscle denervation. As anticipated, as tissue healing stabilized, these functional differences dissipated between 6 and 12-months postoperatively. Importantly, radiographic analysis revealed no significant intergroup differences in HFO or LLD, reinforcing the consensus that short- and long-term joint function is dictated more by prosthesis design, proper cementing techniques, and structured rehabilitation than by the operative window (32–34).

Hematological analysis provided additional insights into surgical trauma. While total transfusion rates did not differ significantly (likely reflecting strict, individualized clinical transfusion thresholds), the evaluation of Hb concentrations told a more nuanced story (35). Expectedly, both groups experienced postoperative Hb drops; however, the Day-1 Hb levels were significantly higher in the SuperCap group. This implies that the SuperCap approach substantially blunts overall blood loss, particularly postoperative occult (hidden) bleeding, owing to minimal tissue disruption. Interestingly, systemic inflammatory markers (CRP and ESR) showed no statistical difference between the two cohorts, suggesting that the systemic stress response remains comparable despite differences in local tissue trauma. Conversely, serum CK levels-a biochemical indicator of acute skeletal muscle injury-painted a distinct picture of local trauma. While both procedures elevated CK levels from baseline, the SuperCap group consistently manifested significantly lower CK concentrations on postoperative Days 1, 3, and 7. This provides objective biochemical corroboration that the true intermuscular dissection of the SuperCap approach intrinsically limits myofiber injury, thereby contributing to the accelerated functional milestones observed in this cohort.

Our findings align with recent robust evidence highlighting the advantages of superior capsular approaches (SuperCap/SuperPATH) in geriatric HA. Comprehensive meta-analyses by Ramadanov et al. demonstrated that these tissue-sparing techniques significantly improve early functional outcomes, notably the short-term HHS, while substantially reducing incision length, intraoperative blood loss, and time to postoperative mobilization (17). Furthermore, a recent network meta-analysis ranked the SuperPATH approach superior to both the DAA and conventional approaches, specifically emphasizing a lower overall complication rate (36). Crucially, meta-regression analyses have identified advanced age (≥70 years) as a primary predictor for these early functional benefits, reinforcing the technique's specific suitability for the frail geriatric demographic (15). This accelerated recovery trajectory is largely attributed to the muscle-sparing nature of the approach, which preserves dynamic pelvic stabilizers and safely facilitates immediate postoperative weight-bearing (16).

At the same time, the SuperCap approach should not be regarded as a universally simple technique. For many surgeons, it represents a new operative concept and is associated with a learning curve during the initial adoption period. In our experience, the early technical challenges were mainly related to intraoperative judgment and team coordination, particularly determining the appropriate length of the preserved femoral neck, achieving smooth reduction through limited superior exposure, and consistently controlling stem anteversion. These issues may be especially relevant during the introductory phase of the technique. Therefore, structured training, appropriate case selection, and gradual adoption are important to ensure procedural safety and reproducibility.

In addition to mitigating musculoskeletal trauma, the SuperCap approach offers significant hemodynamic benefits. Recent comparative literature indicates that HA performed via this approach results in a markedly lower postoperative blood transfusion rate compared to the traditional posterolateral approach, even in frail patients undergoing active antithrombotic therapy (37). This reduction in blood loss and subsequent transfusion requirements is critical for minimizing systemic physiological stress and avoiding associated perioperative complications in elderly patients with femoral neck fractures.

## Limitations

Despite these encouraging findings, this study is subject to several limitations. First, the sample size is relatively small, potentially limiting the statistical power to detect differences in rare adverse events (e.g., dislocation, deep infection). Second, complete blinding was not feasible due to the visible difference in incision length; patient awareness of allocation may have introduced expectation bias affecting patient-reported outcomes (VAS, HHS subcomponents). Third, the 12-month follow-up duration precludes the evaluation of implant survivorship and late-onset complications such as aseptic loosening or osteolysis. Fourth, a formal health economic analysis was not conducted; evaluating whether the clinical benefits offset any potential differences in healthcare resource utilization associated with specialized minimally invasive instrumentation is warranted. Finally, this was a single-center study with all procedures performed by a single experienced surgeon, which may limit generalizability to other settings or surgeons early in their learning curve. Future large-scale, multicenter trials with extended clinical and radiographic follow-up (e.g., >5 years) are warranted to comprehensively establish the longitudinal value of the SuperCap approach. Additionally, future studies should incorporate patient-reported outcome measures (PROMs) beyond HHS, such as the Hip Disability and Osteoarthritis Outcome Score (HOOS) or EQ-5D, to provide a more comprehensive assessment of quality of life. The generalizability of our findings to surgeons with varying levels of experience with the SuperCap technique also requires investigation.

## Conclusion

In conclusion, hip hemiarthroplasty via the SuperCap approach offers clear early clinical advantages over the traditional posterolateral approach for elderly patients with displaced femoral neck fractures. By effectively minimizing skeletal muscle trauma and intraoperative blood loss, the SuperCap technique limits surgical stress, thereby accelerating early postoperative pain relief, ambulation, and hospital discharge. Although mid- to long-term functional outcomes and radiographic implant positioning are comparable between the two approaches, the rapid functional rehabilitation profile of SuperCap makes it a compelling, minimally invasive option for geriatric patients who are particularly vulnerable to bedrest-related complications. Future large-cohort, multicenter studies with extended follow-up are warranted to evaluate the long-term implant survivorship and health-economic benefits of this technique.

## Data Availability

The original contributions presented in the study are included in the article/Supplementary Material, further inquiries can be directed to the corresponding author.
